# Advanced Biomaterials for Restorative Dentistry: From Biocompatibility to Bioactive and Smart Materials

**DOI:** 10.3390/bioengineering13050542

**Published:** 2026-05-09

**Authors:** Maria Claudia Albu, Corina Laura Ștefănescu, Rodica Maria Murineanu, Mircea Grigorian, Liliana Sachelarie, Agripina Zaharia, Loredana Liliana Hurjui, Aureliana Caraiane

**Affiliations:** 1 Faculty of Dentistry, Ovidius University, 900527 Constanta, Romania; claudia.albu@365.univ-ovidius.ro (M.C.A.); corina.stefanescu@365.univ-ovidius.ro (C.L.Ș.); rodica.murineanu@365.univ-ovidius.ro (R.M.M.); mircea.grigorian@365.univ-ovidius.ro (M.G.); aureliana.caraiane@365.univ-ovidius.ro (A.C.); 2 Department of Dental Medicine, Apollonia University, 700511 Iasi, Romania; 3 Private Dental Practice, 900162 Constanta, Romania; agrizaharia@yahoo.com; 4 Faculty of Medicine, Grigore T. Popa University of Medicine and Pharmacy Iasi, University Street 16, 700115 Iasi, Romania; loredana.hurjui@umfiasi.ro

**Keywords:** advanced dental materials, restorative dentistry, dental biomaterials, biocompatibility, bioactive restorative materials, nanotechnology in dentistry, CAD-CAM materials, dental ceramics

## Abstract

(1) Background: The development of advanced dental biomaterials has significantly improved restorative dentistry, shifting the focus from purely mechanical restoration toward materials capable of interacting biologically with oral tissues. Modern restorative materials are expected to demonstrate high biocompatibility, adequate mechanical properties, and potential bioactivity that may support tissue preservation and long-term clinical performance. This review aims to analyze recent advances in next-generation dental restorative materials and to evaluate their biological compatibility and potential clinical relevance. (2) Methods: A narrative literature review was conducted using major scientific databases, including PubMed, Scopus, and Web of Science, focusing on studies addressing advanced polymer-based composites, bioactive restorative materials, dental ceramics, computer-aided design and computer-aided manufacturing (CAD–CAM) restorative systems, and nanostructured biomaterials used in restorative dentistry. Relevant studies published in recent years were analyzed with respect to material composition, biological response, and reported clinical performance. (3) Results: The reviewed literature indicates that modern dental biomaterials, including nanocomposites, bioactive glass-containing materials, calcium silicate–based systems, and hybrid ceramic materials, show improved mechanical stability, enhanced remineralization potential, and reduced bacterial adhesion compared with traditional restorative materials. Advances in nanotechnology and material engineering have also contributed to the development of antimicrobial and bioactive restorative systems. (4) Conclusions: Next-generation dental restorative materials demonstrate promising characteristics that may improve clinical outcomes and biological integration in restorative dentistry; however, further long-term clinical investigations are required to fully confirm their safety, durability, and long-term effectiveness.

## 1. Introduction

Restorative dentistry has undergone remarkable transformation over the past decades due to continuous advances in dental biomaterials and material engineering. Traditionally, restorative materials such as dental amalgam, glass ionomer cements, and early resin-based composites were primarily designed to restore the mechanical integrity of damaged teeth [1]. However, modern dentistry increasingly demands materials that combine high mechanical performance with biological compatibility, aesthetic integration, and long-term stability within the complex oral environment [1,2]. The oral cavity presents unique challenges for restorative materials because they must withstand mechanical loading, thermal fluctuations, enzymatic degradation, and continuous exposure to saliva and the oral microbiota [2,3].

Recent studies have demonstrated that the oral environment is a highly dynamic system in which mechanical, chemical, enzymatic, and microbiological factors interact simultaneously, significantly influencing the degradation and clinical performance of restorative materials [4,5,6].

In recent years, the development of advanced dental biomaterials has shifted the paradigm from passive restorative materials toward bioactive and multifunctional systems capable of interacting with biological tissues and promoting tissue repair or remineralization [7]. Bioactive restorative materials, including ion-releasing composites and bioactive glass-based systems, can release therapeutic ions such as calcium, phosphate, and fluoride, thereby contributing to enamel and dentin remineralization while potentially inhibiting bacterial colonization [8]. These innovations aim to extend the longevity of restorations and reduce the risk of secondary caries, which remains a leading cause of restoration failure.

Recent literature has emphasized the transition toward therapeutic and bioactive restorative materials, which actively participate in remineralization processes and modulation of the oral microenvironment [8,9].

Another significant area of progress in dental biomaterials is the development of high-performance ceramics and hybrid materials for digital dentistry and CAD–CAM restorative systems. Materials such as zirconia and lithium disilicate ceramics demonstrate excellent mechanical strength, optical properties, and clinical durability, making them widely used for crowns, veneers, and implant-supported restorations [1,10]. Simultaneously, nanotechnology has enabled the development of nanocomposites and nanofilled restorative materials that exhibit improved wear resistance, enhanced polishability, and better marginal adaptation compared with conventional composites [4].

Advances in nanotechnology and materials engineering have also enabled the incorporation of antimicrobial agents and bioactive fillers into restorative materials, thereby improving resistance to bacterial adhesion and biofilm formation [10,11,12].

Despite these advancements, several controversies and challenges remain in the field of dental biomaterials. While bioactive restorative materials have demonstrated promising biological interactions with dental tissues, questions remain regarding their long-term clinical stability, degradation behavior, and potential cytotoxicity associated with certain nanoparticles or ion-release systems. Furthermore, polymerization shrinkage, marginal leakage, and mechanical fatigue remain important limitations affecting the longevity of resin-based restorative materials [1,5].

Moreover, the interaction between restorative materials and the oral microbiota has emerged as a key factor influencing the longevity of restorations, particularly through biofilm formation and secondary caries development [12,13,14].

Therefore, continuous investigation of material composition, biological interactions, and clinical performance is necessary to guide evidence-based dental practice.

Recent interdisciplinary approaches integrating advanced materials science, nanotechnology, and digital dentistry have further expanded the potential of restorative materials, supporting the development of more durable and biologically integrated therapeutic solutions [9,10,11,12,13,14].

Although numerous studies have investigated individual classes of dental biomaterials, a comprehensive, integrative analysis correlating bioactivity, nanostructure, antimicrobial properties, and digital manufacturing approaches remains limited in the current literature. Unlike previous reviews that primarily analyze individual classes of dental biomaterials in isolation, the present work provides a clinically oriented integrative framework that correlates bioactivity, nanostructure, antimicrobial functionality, and digital manufacturing technologies.

In addition, this review emphasizes the translational gap between experimental biomaterials and clinically validated systems, highlighting the discrepancy between laboratory innovation and real-world dental practice—an aspect that remains insufficiently addressed in current literature. Furthermore, it highlights emerging trends in smart biomaterials and interdisciplinary approaches, offering a structured synthesis of recent developments and identifying future research directions in restorative dentistry.

Given the rapid evolution of dental biomaterials and their increasing complexity, a comprehensive evaluation of next-generation restorative materials is essential. The aim of this review is to analyze recent advances in advanced dental biomaterials used in restorative dentistry, with particular emphasis on their biocompatibility, bioactivity, and emerging clinical applications [8,14,15,16,17,18,19,20].

Understanding the interactions between these materials and the oral cavity’s biological environment may contribute to developing more durable, biologically integrated restorative strategies in modern dental practice.

## 2. Materials and Methods

### 2.1. Study Design

This study was designed as a narrative literature review aimed at synthesizing current scientific evidence regarding advanced dental biomaterials used in restorative dentistry. The review focuses on recent developments in bioactive restorative materials, nanostructured composites, dental ceramics, and CAD–CAM restorative systems, with particular attention to their biocompatibility, bioactivity, mechanical performance, and clinical applicability.

### 2.2. Literature Search Strategy

The objective of the review was to analyze contemporary research describing the composition, biological interaction, and clinical performance of next-generation restorative materials and to highlight emerging directions in dental biomaterials research.

A focused literature search was conducted using several major scientific databases, including PubMed/MEDLINE, Scopus, Web of Science, and Google Scholar, as well as complementary sources. The search strategy was designed to identify relevant studies addressing recent advances in dental restorative biomaterials. Combinations of keywords such as “dental biomaterials”, “restorative dentistry”, “bioactive dental materials”, “nanocomposites in dentistry”, “bioactive glass dental materials”, “dental ceramics”, “CAD–CAM restorative materials”, and “biocompatibility of dental materials” were used to retrieve relevant publications.

### 2.3. Inclusion and Exclusion Criteria

The selection of studies followed predefined inclusion and exclusion criteria in order to ensure the scientific relevance and quality of the analyzed literature. Publications were included if they were peer-reviewed scientific articles investigating dental restorative biomaterials, including studies addressing material composition, biological properties, mechanical behavior, antimicrobial activity, or clinical performance. Systematic reviews, meta-analyses, and experimental research on modern restorative materials, including resin-based composites, bioactive restorative materials, dental ceramics, and CAD–CAM restorative systems, were also considered eligible. Only articles written in English and available in full text were included in the analysis.

Studies were excluded if they were not directly related to restorative dental biomaterials, lacked sufficient methodological description, or represented conference abstracts without complete scientific data. In addition, publications with limited relevance to restorative dentistry or those focusing exclusively on unrelated dental fields were not considered. Priority was given to studies published between 2010 and 2025 in order to capture the most recent developments in dental biomaterials research, while earlier landmark publications were included when necessary to clarify fundamental concepts in biomaterial science.

### 2.4. Data Extraction and Synthesis

Relevant information from the selected publications was systematically extracted and organized according to the main thematic areas related to restorative dental biomaterials. The extracted data included aspects such as material composition, biological properties, mechanical performance, antimicrobial activity, and reported clinical applications. Particular attention was given to studies describing bioactive restorative materials, resin-based composites, dental ceramics, hybrid restorative materials, and CAD–CAM systems. The collected information was analyzed qualitatively and synthesized in order to highlight the most important developments in the field, as well as current trends in the design and clinical application of advanced dental restorative biomaterials.

## 3. Results

### 3.1. Resin-Based Composite Biomaterials

**Figure 1:**
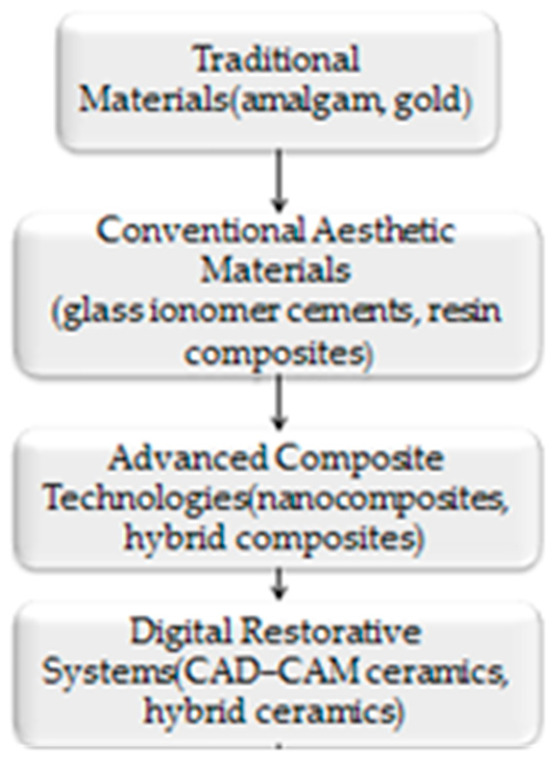
Schematic representation of the evolution of restorative dental biomaterials, illustrating the transition from traditional metallic restorations to modern bioactive and smart materials.

Resin-based composite materials represent one of the most widely used restorative options in contemporary dentistry due to their favorable aesthetic properties, acceptable mechanical strength, and improved adhesive capabilities [6]. These materials consist primarily of a polymeric organic matrix, inorganic filler particles, and a silane coupling agent that ensures adhesion between the two phases [7,8,9]. Over the past decades, continuous improvements in filler technology and resin chemistry have led to the development of nanocomposites, nanohybrid composites, and bulk-fill materials, which demonstrate enhanced mechanical performance and improved handling properties [8,9,10], Figure 1.

Recent research has focused on optimizing filler particle size, distribution, and surface treatment in order to improve wear resistance, reduce polymerization shrinkage, and increase long-term clinical durability [9]. In particular, nanotechnology has played an essential role in improving the physical and mechanical properties of composite resins, enabling better polishability, enhanced aesthetic integration, and greater resistance to marginal degradation [10]. Table 1 summarizes the main categories of restorative dental biomaterials and their principal characteristics.

**Figure 2:**
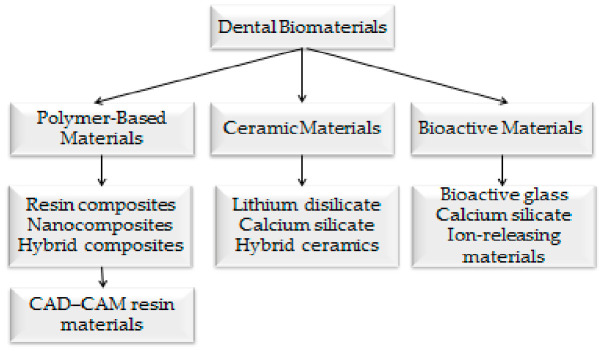
Classification of modern dental biomaterials.

Figure 1 and Figure 2 illustrate the technological evolution and classification of contemporary restorative biomaterials.

However, despite significant improvements in material composition and performance, resin-based composites still present limitations such as polymerization shrinkage, long-term degradation, and the potential cytotoxic effects of residual monomers and degradation products, which may affect both their clinical longevity and interaction with surrounding tissues. Moreover, discrepancies between laboratory performance and clinical outcomes remain a challenge.

### 3.2. Bioactive Restorative Materials

In recent years, the concept of bioactivity has gained significant attention in restorative dentistry, reflecting a shift from inert restorative materials toward systems capable of interacting with biological tissues. Bioactive dental materials are designed not only to restore the structural integrity of the tooth but also to promote biological responses such as remineralization, antibacterial activity, and improved integration with surrounding tissues [11].

One of the most widely investigated groups of bioactive restorative materials includes those based on bioactive glass and calcium silicate compounds. These materials have the ability to release biologically active ions such as calcium, phosphate, and fluoride, which contribute to the formation of hydroxyapatite and support the remineralization of dental hard tissues [12]. The release of these ions can also create a local environment that is less favorable for bacterial proliferation, thereby reducing the risk of secondary caries [13,14,15].

Calcium silicate–based materials, including mineral trioxide aggregate (MTA) and newer bioactive cements, have demonstrated promising biological properties such as biocompatibility, bioactivity, and the ability to stimulate mineralized tissue formation. These materials are widely used in various clinical procedures, including pulp capping, root repair, and regenerative endodontic treatments [14,15,16,17].

Another important direction in the development of bioactive restorative materials involves incorporating ion-releasing fillers and antimicrobial agents into resin-based composite systems. These modifications aim to combine the aesthetic and mechanical advantages of composite resins with additional therapeutic functions, such as the release of fluoride, calcium, or phosphate ions that contribute to the prevention of demineralization and the promotion of remineralization [15].

Recent advances in biomaterial science have led to the development of smart restorative materials, designed to actively interact with the oral environment and respond to specific biological or physicochemical stimuli. Unlike conventional restorative materials, which are largely passive once placed in the oral cavity, these innovative biomaterials can respond dynamically to environmental changes, such as fluctuations in pH, bacterial metabolic activity, or ionic concentrations within the surrounding dental tissues [16].

One of the most important characteristics of these materials is their ability to respond to acidic conditions typically associated with cariogenic bacterial activity, thereby supporting remineralization processes and reducing the progression of demineralization [17,18,19,20]. Additionally, certain smart restorative systems incorporate antimicrobial agents or bioactive fillers that disrupt bacterial biofilms, thereby reducing the risk of secondary caries and improving the long-term stability of dental restorations.

**Figure 3:**
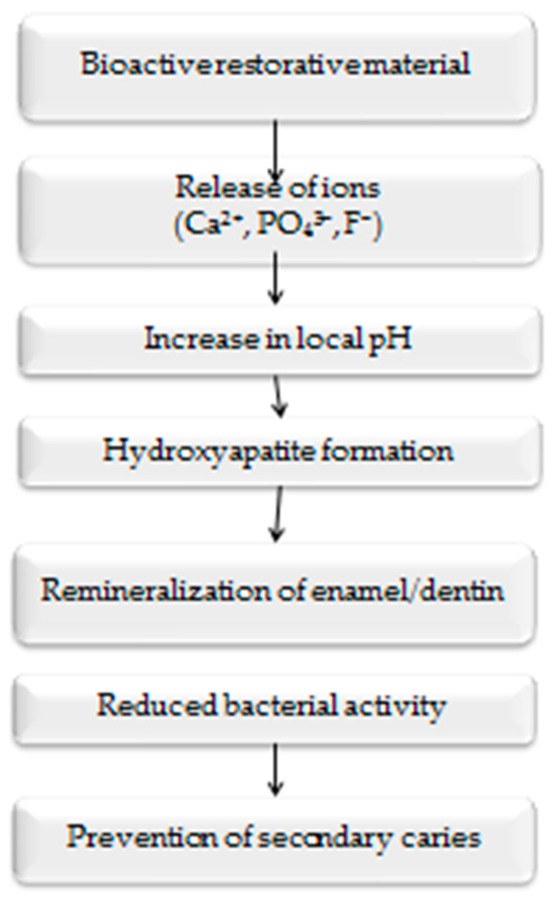
Schematic illustration of the bioactivity mechanism of modern restorative dental materials, including ion release, hydroxyapatite formation, remineralization of dental tissues, and antibacterial effects.

These responsive materials often integrate pH-sensitive nanoparticles, ion-releasing glass fillers, or bioactive ceramic components, which allow the material to adapt its behavior according to the surrounding microenvironment. Such technologies represent an important step toward the development of therapeutic restorative materials, capable not only of replacing lost dental tissues but also of actively supporting biological repair processes and maintaining oral health [18]. Consequently, smart biomaterials are increasingly viewed as a key component of the emerging paradigm of minimally invasive and biologically driven restorative dentistry. The biological interactions associated with bioactive restorative materials involve complex processes including ion release, hydroxyapatite formation, and the remineralization of dental tissues. These mechanisms contribute to the prevention of secondary caries and improved long-term restoration performance. The main stages of bioactivity in restorative dental materials are illustrated in Figure 3.

The main categories of bioactive restorative materials and their mechanisms of action, including ion release, remineralization potential, and antimicrobial activity, are summarized in Table 2.

Although bioactive materials show considerable potential and promising biological interactions, their long-term clinical effectiveness, stability under functional conditions, and biocompatibility under physiological conditions remain insufficiently documented. This highlights a significant gap between experimental research and routine clinical application.

### 3.3. Dental Ceramics and Hybrid Materials

Dental ceramics have become an essential component of contemporary restorative dentistry due to their excellent aesthetic properties, high biocompatibility, and superior mechanical performance compared with many conventional restorative materials. These materials are widely used in indirect restorations such as crowns, veneers, inlays, and onlays, providing long-term durability and optimal aesthetic integration with natural dentition [19].

Modern dental ceramics include several categories, among which feldspathic ceramics, lithium disilicate glass-ceramics, and zirconia-based ceramics are the most commonly used. Lithium disilicate ceramics, in particular, have gained widespread popularity for their combination of high flexural strength, translucency, and excellent optical properties, which enable restorations to closely mimic the natural appearance of dental tissues [20]. Zirconia ceramics, on the other hand, are characterized by exceptional fracture resistance and mechanical stability, making them suitable for high-load restorations such as posterior crowns and implant-supported prostheses [21,22,23,24].

In recent years, the development of hybrid ceramic materials has represented an important advancement in restorative biomaterials. These materials combine ceramic networks with polymer components, resulting in structures that exhibit improved elasticity, reduced brittleness, and enhanced fracture resistance compared with traditional ceramics. Hybrid ceramics, such as polymer-infiltrated ceramic networks (PICN), provide a balance between mechanical strength and flexibility, allowing them to better absorb occlusal forces and reduce the risk of catastrophic failure [22,23,24,25,26,27,28].

Another major innovation in this field is the integration of CAD–CAM technology in the fabrication of ceramic and hybrid restorations. Computer-aided design and computer-aided manufacturing systems enable highly precise and reproducible restorations, improving marginal adaptation and reducing laboratory processing time. CAD–CAM materials include zirconia blocks, lithium disilicate ceramics, and resin–ceramic hybrid materials specifically designed for digital milling systems [23,29].

Overall, dental ceramics and hybrid materials represent a significant advancement in restorative dentistry, offering improved mechanical performance, aesthetic outcomes, and compatibility with modern digital workflows. Their continued development reflects the growing emphasis on durable, minimally invasive, and aesthetically optimized restorative solutions. The main categories of dental ceramic and hybrid restorative materials and their principal characteristics are summarized in Table 3.

While ceramic materials demonstrate excellent mechanical properties, their brittleness and technique sensitivity remain important clinical limitations, particularly in high-stress environments. Although they are generally considered highly biocompatible, factors related to material processing and surface treatments may influence their biological response.

### 3.4. CAD–CAM Materials in Digital Restorative Dentistry

Computer-aided design and computer-aided manufacturing (CAD–CAM) technologies have significantly transformed modern restorative dentistry by enabling the fabrication of highly precise and reproducible dental restorations. These digital systems enable clinicians to design and manufacture indirect restorations using advanced biomaterials that offer improved mechanical properties, aesthetic performance, and long-term durability. The integration of digital workflows has improved treatment efficiency while reducing laboratory processing time and minimizing potential human error during restoration fabrication [23].

The CAD–CAM restorative workflow generally involves three main stages: digital scanning, computer-assisted design, and automated manufacturing of the restoration. Intraoral scanners are used to capture high-resolution three-dimensional images of the prepared tooth structures, which are subsequently processed using specialized software to design the restoration geometry. The final restoration is then milled or manufactured from prefabricated biomaterial blocks using computer-controlled milling units [24,30,31,32,33].

A wide range of materials has been specifically developed for CAD–CAM restorative systems. These include zirconia ceramics, lithium disilicate glass-ceramics, hybrid ceramics, and resin nanoceramic materials. Zirconia is widely recognized for its exceptional mechanical strength, fracture resistance, and long-term clinical stability, making it particularly suitable for posterior crowns and implant-supported prostheses [19,30,34,35,36,37]. Lithium disilicate ceramics, in contrast, offer superior translucency and optical properties, allowing the fabrication of highly aesthetic restorations that closely mimic natural dental tissues [20,30,34,35,36].

Hybrid CAD–CAM materials represent another important advancement in restorative biomaterials. These materials combine ceramic and polymer networks, resulting in structures that exhibit improved elasticity and reduced brittleness compared with traditional ceramics. Polymer-infiltrated ceramic network (PICN) materials, for example, demonstrate enhanced shock absorption and resistance to crack propagation, making them suitable for minimally invasive restorative procedures [22,37,38].

Overall, CAD–CAM restorative systems represent a major milestone in digital dentistry, enabling the fabrication of highly accurate restorations while improving clinical efficiency and patient outcomes. The continuous development of CAD–CAM compatible biomaterials and digital technologies is expected to further expand the possibilities of digital restorative dentistry and contribute to more predictable and personalized dental treatments [24,28,39].

The main mechanical properties of ceramic and hybrid restorative materials reported in the literature are summarized in Table 4.

The biocompatibility of CAD–CAM materials is generally favorable; however, factors such as surface finishing and bonding protocols may influence their interaction with oral tissues. In clinical practice, these materials must demonstrate not only mechanical strength and precision but also long-term stability under physiological conditions, including exposure to occlusal forces, thermal cycling, and oral fluids. Studies have shown that materials such as zirconia and lithium disilicate exhibit favorable in vivo performance, with high survival rates and good resistance to wear and fracture [30,34,35]. Nevertheless, the clinical success of CAD–CAM restorations is also influenced by factors such as material thickness and patient-specific conditions, highlighting the importance of appropriate material selection and clinical technique [36].

### 3.5. Nanostructured and Smart Dental Biomaterials, Digital Technologies and Artificial Intelligence

**Figure 4:**
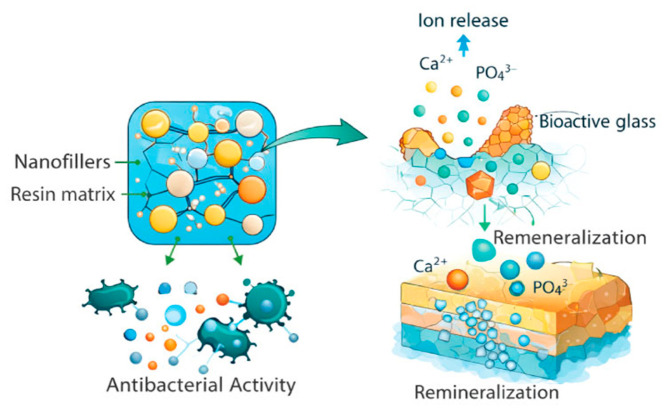
Schematic illustration of nanostructured and bioactive restorative materials, highlighting filler–matrix interactions, ion release mechanisms, and their role in remineralization and antibacterial activity.

Recent advances in nanotechnology have significantly influenced the development of modern dental restorative materials. The incorporation of nanoscale filler particles and nanostructured components into restorative biomaterials has led to improved mechanical properties, enhanced surface characteristics, and better biological interactions with dental tissues. Nanocomposite materials, for instance, offer improved polishability, increased wear resistance, and enhanced aesthetic integration with natural dentition, making them widely used in contemporary restorative dentistry [24,31] (Figure 4).

Nanostructured biomaterials enhance the interfacial bonding between filler particles and the organic resin matrix. Surface modification of nanoparticles, particularly through silane coupling agents, improves the compatibility between inorganic fillers and polymer matrices, resulting in increased mechanical stability and reduced degradation in the oral environment. These developments have significantly improved the functional performance of restorative materials while maintaining favorable aesthetic and handling properties [25,31].

Beyond mechanical enhancement, nanotechnology has enabled the development of smart restorative materials that can dynamically interact with the oral environment. Unlike conventional materials, which remain largely passive after placement, these advanced systems are designed to respond to physicochemical stimuli, including pH fluctuations, bacterial metabolic activity, and changes in ionic concentration within dental tissues. Under acidic conditions associated with cariogenic biofilms, these materials can release therapeutic ions, including calcium, phosphate, and fluoride, thereby promoting remineralization and reducing the risk of secondary caries [26,32].

Among these approaches, pH-responsive biomaterials represent a particularly important advancement. These systems are engineered to detect acidic environments generated by bacterial metabolism and to trigger the controlled release of ions, contributing to the maintenance of mineral balance in enamel and dentin. The incorporation of pH-sensitive nanofillers and acid-triggered ion-release mechanisms into modern composite systems allows restorative materials to actively participate in preventing demineralization processes [26,32]. However, their long-term in vivo performance remains an area of ongoing investigation, as factors such as material stability, ion-release kinetics, and resistance to biofilm formation may influence their clinical effectiveness [33].

In addition to remineralizing capabilities, antimicrobial strategies have been increasingly integrated into restorative biomaterials. The incorporation of antimicrobial nanoparticles or bioactive fillers can inhibit bacterial adhesion and biofilm formation on restorative surfaces, which is essential for preventing recurrent caries and restoration failure. Various approaches have been explored, including nanoparticles such as silver and zinc oxide, as well as antibacterial monomers like quaternary ammonium compounds, which enhance antimicrobial performance while preserving mechanical and aesthetic properties [27,33]. These strategies may contribute to improving the long-term stability of dental restorations by reducing bacterial colonization and biofilm development [28,33].

In parallel with advances in nanotechnology, digital technologies have profoundly transformed restorative dentistry. Computer-aided design and computer-aided manufacturing (CAD–CAM) systems enable the fabrication of highly precise restorations using advanced ceramic, hybrid, and resin-based biomaterials. These digital workflows improve accuracy, reproducibility, and efficiency, while allowing better control over material microstructure and mechanical performance [23,34].

More recently, artificial intelligence (AI) has emerged as a promising tool in dental biomaterials research and digital dentistry. Machine learning algorithms can analyze large datasets to predict mechanical properties, optimize material compositions, and simulate biomaterial performance under clinical conditions. These AI-driven approaches have the potential to accelerate the development of next-generation restorative materials with improved durability, bioactivity, and biocompatibility, while also supporting personalized treatment planning based on patient-specific parameters [30,35,36].

The main emerging technological approaches and biomaterial strategies currently investigated in restorative dentistry, together with their advantages and available clinical evidence, are summarized in Table 5.

Overall, the integration of nanotechnology, smart biomaterials, digital manufacturing technologies, and artificial intelligence represents a major step toward the development of next-generation restorative materials. These innovations aim not only to restore lost dental structures but also to interact with the biological environment, enhance the stability of long-term restorations, and support the maintenance of oral health through multifunctional therapeutic mechanisms [28,29,30,36].

Despite promising experimental results, the clinical translation of nanostructured and AI-assisted biomaterials remains limited, and further validation through long-term clinical trials is required.

## 4. Discussion

The continuous evolution of dental restorative materials reflects the growing demand for biomaterials that combine mechanical durability, aesthetic integration, and biological compatibility. Over the past decades, substantial progress has been made in the development of resin-based composites, bioactive restorative materials, advanced ceramics, and CAD–CAM compatible restorative materials. Each category offers specific advantages and limitations, and their appropriate selection depends largely on the clinical context and functional requirements of the restoration [29].

A relevant aspect of evaluating advanced dental biomaterials is comparing conventional restorative materials with emerging bioactive or nanostructured systems. Conventional resin composites primarily provide mechanical restoration of dental tissues, whereas bioactive composites and nanostructured materials may additionally promote remineralization and biological interaction with the surrounding tissues. Studies have shown that bioactive restorative materials capable of releasing calcium and phosphate ions may contribute to inhibiting demineralization processes and improving the stability of the tooth–restoration interface [24,31,38].

Resin-based composites remain among the most widely used restorative materials due to their excellent aesthetic properties and minimally invasive application techniques. Advances in filler technology, particularly the introduction of nanofillers and nanohybrid composites, have significantly improved the mechanical performance and wear resistance of these materials [11,24]. Nevertheless polymerization shrinkage, marginal degradation, and long-term mechanical stability remain important challenges that may influence restoration longevity [30].

Bioactive restorative materials represent an important step toward a more biologically oriented approach in restorative dentistry. Materials capable of releasing therapeutic ions, such as calcium, phosphate, and fluoride, can actively contribute to remineralization processes and inhibit bacterial activity in the oral environment. Bioactive glass and calcium silicate-based materials, in particular, have demonstrated promising properties in promoting mineralized tissue formation and supporting the regeneration of dental structures. However, some bioactive restorative materials may still have limitations in mechanical strength and long-term durability, which may restrict their use in high-load restorative situations [31].

Ceramic materials, especially lithium disilicate and zirconia-based ceramics, have become fundamental components of modern restorative dentistry due to their excellent mechanical strength and superior aesthetic properties. Zirconia ceramics provide exceptional fracture resistance and are widely used in posterior restorations and implant-supported prostheses. Lithium disilicate ceramics, on the other hand, offer improved translucency and optical properties that allow restorations to closely mimic natural dental tissues. Despite these advantages, ceramics remain relatively brittle materials and may require careful preparation design and adhesive protocols to minimize the risk of fracture [30,32,33,34,35,36,37].

A significant gap persists between experimental research on advanced biomaterials and their clinical validation, as many promising materials have not yet been evaluated through long-term randomized clinical trials. In addition, important discrepancies remain between newly developed experimental systems and commercially available materials used in clinical practice. While research often focuses on innovative compositions with enhanced bioactivity or antimicrobial properties, clinically approved materials are primarily selected based on proven mechanical reliability, ease of use, and long-term performance. This highlights the critical need for translational research to bridge the gap between laboratory findings and real-world clinical outcomes.

Despite significant advances in material design, the clinical translation of many bioactive and nanostructured systems remains limited. In real-world practice, material selection continues to prioritize mechanical reliability and long-term survival over experimental bioactivity, thereby highlighting a persistent gap between laboratory innovation and clinical applicability.

A critical aspect of evaluating advanced dental biomaterials is balancing trade-offs among material properties. While bioactive materials offer the advantage of promoting remineralization and biological interaction, they may exhibit reduced mechanical strength and long-term durability compared with conventional restorative materials [31,33]. Similarly, the incorporation of antimicrobial agents may enhance resistance to bacterial colonization but can potentially affect material stability or biocompatibility [27,33].

These trade-offs highlight the complexity of designing multifunctional restorative materials and emphasize the need for optimizing material performance according to specific clinical requirements.

The safety and biocompatibility of dental restorative materials represent critical factors for their clinical application. Although modern biomaterials are designed to minimize adverse biological effects, concerns remain regarding the potential cytotoxicity of certain components, particularly nanoparticles, monomers, and ion-releasing systems [9,10]. The release of residual monomers and degradation products may induce local inflammatory responses or affect surrounding tissues, highlighting the importance of material stability and controlled ion release. Furthermore, the long-term biological impact of nanostructured and bioactive materials under physiological conditions remains insufficiently understood, emphasizing the need for further in vivo and clinical studies to ensure their safety and biocompatibility [13,14]. This aspect further reinforces the gap between experimental developments and their safe translation into clinical practice.

Several recent review articles have explored the development of dental biomaterials, focusing primarily on their composition, mechanical properties, and clinical performance [1,2,3]. For instance, Woźniak-Budych et al. [1] emphasized the importance of biocompatibility in dental materials, while Abozaid et al. [2] and Musani et al. [3] provided comprehensive overviews of bioactive restorative systems and their mechanisms of action. However, these studies tend to analyze material categories individually, without fully integrating the interactions between bioactivity, nanostructure, antimicrobial properties, and digital fabrication techniques.

In addition, previous reviews have largely focused on experimental and material-based perspectives, with limited emphasis on the discrepancies between laboratory findings and real-world clinical performance. While numerous studies report promising in vitro results for bioactive and nanostructured materials, their long-term clinical validation remains insufficiently documented [4,5,6]. This highlights a persistent gap between research innovation and routine clinical application, particularly for commercially available restorative materials.

The present review contributes to the existing literature by providing a more integrative and multidisciplinary perspective that correlates material properties with biological interactions and clinical applicability. By combining insights from bioactive materials, nanotechnology, antimicrobial strategies, and digital dentistry, this work offers a more comprehensive understanding of the current state and future direction of restorative biomaterials.

The rapid expansion of CAD–CAM technologies has further transformed restorative dentistry by enabling the fabrication of highly precise restorations using standardized biomaterial blocks. CAD–CAM compatible materials offer improved consistency in microstructure and mechanical properties compared with conventionally fabricated restorations. Hybrid materials, such as polymer-infiltrated ceramic networks, attempt to combine the strength of ceramics with the elasticity of polymer-based materials, thereby improving fracture resistance and reducing brittleness. Nevertheless, the long-term clinical performance of some newer CAD–CAM materials still requires further investigation through long-term clinical studies [33,38,39,40,41].

Digital restorative materials manufactured using CAD–CAM technologies, such as lithium disilicate ceramics, zirconia-based ceramics, and hybrid resin–ceramic materials, have significantly improved the precision and durability of indirect restorations. While ceramic materials generally exhibit excellent mechanical strength and long-term clinical stability, hybrid materials offer improved stress absorption and easier repair. Therefore, the selection of CAD–CAM materials often depends on the clinical situation, functional demands, and aesthetic requirements [23,34,37]. Beyond material composition and digital fabrication techniques, the long-term clinical performance of restorative materials remains a fundamental aspect in evaluating their success.

Clinical longevity remains a critical factor in the evaluation of restorative materials. Long-term clinical studies indicate that modern resin composites and ceramic-based restorations can achieve 85–90% survival rates over 10 years. However, secondary caries, material degradation, and interface failure remain common causes of restoration replacement. The development of smart and bioactive restorative materials capable of reducing bacterial colonization and promoting remineralization may contribute to improving the long-term stability of dental restorations [38,39,40].

These observations are consistent with findings reported in systematic reviews and long-term clinical studies, which emphasize variability in clinical performance depending on material type, clinical indication, and follow-up duration [32,33,34].

Nanotechnology has opened new perspectives for the development of multifunctional restorative materials. Nanostructured fillers enhance the mechanical properties and polishability of restorative materials and enable the incorporation of bioactive and antimicrobial agents. The concept of smart restorative materials that respond to environmental stimuli represents a promising direction for future research. Such materials may potentially release therapeutic agents in response to pH changes associated with cariogenic activity, contributing to the prevention of secondary caries and restoration failure [34].

Despite these significant advances, several challenges remain in the development of next-generation dental biomaterials. Long-term clinical stability, resistance to biofilm formation, and mechanical reliability under complex oral conditions remain important research priorities. Additionally, the interaction between restorative materials and the oral microbiome warrants further investigation to better understand the biological performance of restorative biomaterials in vivo [30,42,43,44].

Looking toward the future, artificial intelligence (AI) and machine learning technologies may play an increasingly important role in the development and optimization of dental biomaterials. AI-driven materials design has already demonstrated the potential to accelerate the discovery of new material compositions and to predict mechanical and biological performance using large datasets. In restorative dentistry, AI may assist not only in digital treatment planning but also in the development of biomaterials with optimized microstructure, improved mechanical performance, and enhanced biological compatibility [35,44].

Despite the promising developments in advanced dental biomaterials, several limitations remain in the current body of research. Many studies investigating nanostructured and smart biomaterials are conducted in vitro, which may not fully reproduce the complex biological and mechanical conditions of the oral environment. Furthermore, long-term clinical trials evaluating the durability, bioactivity, and antimicrobial effects of these materials are still limited.

Future research should focus on developing multifunctional restorative materials that combine mechanical strength, antimicrobial activity, and bioactive properties. In addition, the integration of artificial intelligence and computational materials design may accelerate the discovery of new biomaterials with optimized mechanical and biological performance. Personalized restorative approaches based on patient-specific biological and functional parameters may also represent an important direction for the future of restorative dentistry.

## 5. Conclusions

The continuous development of dental restorative materials has led to significant improvements in the quality, durability, and biological compatibility of modern restorative treatments. Advances in resin-based composites, bioactive materials, ceramic systems, and CAD–CAM-compatible materials have considerably expanded the therapeutic possibilities available in contemporary dentistry, allowing clinicians to better balance functional performance with aesthetic outcomes.

Among these, resin-based composites continue to be widely used due to their favorable aesthetic properties and minimally invasive application techniques. At the same time, bioactive restorative materials have introduced a new paradigm by actively participating in remineralization processes and contributing to the prevention of secondary caries. In parallel, ceramic systems such as lithium disilicate and zirconia remain reliable options for indirect restorations, owing to their superior mechanical strength and excellent optical properties.

Despite these advances, important challenges persist. The long-term clinical performance of bioactive and nanostructured materials remains insufficiently validated, and discrepancies between in vitro results and in vivo behavior continue to limit the direct translation of experimental findings into clinical practice. In addition, resistance to bacterial colonization, material degradation under complex oral conditions, and the influence of clinical protocols remain critical factors that affect restoration longevity. Consequently, the success of restorative treatments depends not only on material properties but also on appropriate material selection and precise clinical application.

Future developments in restorative dentistry are expected to focus on designing multifunctional biomaterials that simultaneously provide mechanical strength, bioactivity, and antimicrobial activity. Increasing attention should be directed toward long-term in vivo investigations and well-designed clinical trials, which are essential for validating the safety, durability, and biological performance of emerging materials under physiological conditions.

Moreover, the integration of nanotechnology and digital manufacturing approaches, particularly CAD–CAM systems, offers significant potential for improving the structural consistency and functional performance of restorative materials. The development of smart and responsive biomaterials, capable of adapting to the dynamic conditions of the oral environment, represents another promising direction, with the potential to enhance both preventive and therapeutic outcomes.

In addition, the growing role of artificial intelligence and advanced computational methods is likely to influence the future of biomaterial design. Through predictive modeling, optimization of material properties, and support for personalized treatment planning, these technologies may contribute to more efficient and biologically integrated restorative solutions.

Overall, the future of restorative dentistry lies in the convergence of advanced biomaterials, nanotechnology, digital manufacturing, and artificial intelligence, paving the way toward more durable, adaptive, and patient-specific restorative strategies.

## Figures and Tables

**Table 1:** Comparative Overview of Major Restorative Dental Biomaterials.

Material Type	Main Composition	Key Advantages	Limitations	Typical Clinical Applications	Clinical Relevance	References
Amalgam	Silver, tin, copper alloy with mercury	High durability, good compressive strength, long clinical history	Poor aesthetics, environmental concerns, limited adhesion	Posterior restorations (historically common)	Suitable for posterior restorations where durability is prioritized over aesthetics, although its use has declined due to aesthetic and environmental concerns	[1,5]
Glass Ionomer Cement (GIC)	Fluoroaluminosilicate glass + polyacrylic acid	Fluoride release, chemical adhesion to tooth structure, biocompatibility	Lower mechanical strength, wear susceptibility	Cervical lesions, pediatric dentistry, temporary restorations	Recommended for cervical lesions and pediatric applications due to fluoride release and chemical adhesion, particularly in low-stress areas	[1,13]
Resin-Based Composites	Organic resin matrix (Bis-GMA, UDMA) + inorganic fillers	Excellent aesthetics, strong adhesion, minimally invasive preparation	Polymerization shrinkage, technique sensitivity	Direct restorations in anterior and posterior teeth	Preferred for aesthetic and minimally invasive restorations; widely used in both anterior and posterior regions depending on load conditions	[6,11]
Nanocomposites	Resin matrix with nanoscale filler particles	Improved polishability, enhanced mechanical strength, better wear resistance	Technique sensitive, higher cost	Highly aesthetic restorations	Indicated for restorations requiring superior aesthetics and improved surface properties, particularly in visible areas	[8,13]
Bioactive restorative materials	Calcium silicates, bioactive glass, ion-releasing components	Promote remineralization, antimicrobial potential, improved tissue interaction	Limited long-term clinical data	Regenerative and minimally invasive dentistry	Promising for preventive and remineralizing strategies, especially in patients with high caries risk, although long-term clinical validation is still needed	[2,3,14]
Dental Ceramics	Feldspathic porcelain, lithium disilicate, zirconia	Excellent aesthetics, high strength, biocompatibility	Brittle behavior, higher cost, laboratory procedures required	Crowns, veneers, inlays/onlays	Ideal for indirect restorations requiring high aesthetics and mechanical strength, particularly in long-term rehabilitations	[19,20]
CAD–CAM Materials	Hybrid ceramics, resin nanoceramics, zirconia blocks	High precision, digital workflow compatibility, reproducibility	Equipment cost, learning curve	Indirect restorations fabricated digitally	Recommended for digitally fabricated restorations with high precision and reproducibility, especially in modern digital workflows	[6,8]

**Table 2:** Major Bioactive Restorative Materials Used in Contemporary Dentistry.

Material Type	Main Components	Bioactive Mechanism	Clinical Applications	Key References
Bioactive Glass-Based Materials	SiO_2_–CaO–Na_2_O–P_2_O_5_ glass particles	Release of Ca^2+^ and PO_4_^3−^ ions leading to hydroxyapatite formation	Remineralizing restorative materials, dentin repair	[11,12]
Calcium Silicate Cements (e.g., MTA)	Tricalcium silicate, dicalcium silicate, calcium aluminate	Induce mineralized tissue formation and dentin bridge development	Pulp capping, root-end filling, endodontic repair	[14,15,16,17]
Fluoride-Releasing Restorative Materials	Glass ionomer matrix or resin-modified composites with fluoride fillers	Continuous fluoride ion release enhancing enamel remineralization	Cervical lesions, pediatric dentistry	[13]
Ion-Releasing Composite Resins	Resin matrix with calcium phosphate or bioactive glass fillers	Release of Ca^2+^ and PO_4_^3−^ ions to promote remineralization	Direct restorations with preventive effect	[15,16,17]
Smart Bioactive restorative materials	pH-responsive nanoparticles and ion-releasing fillers	Stimulus-responsive ion release under acidic conditions	Prevention of secondary caries, minimally invasive dentistry	[16,17,18]

**Table 3:** Major Dental Ceramic and Hybrid Restorative Materials Used in Contemporary Dentistry.

Material Type	Main Composition	Mechanical Properties	Advantages	Clinical Applications	References
Feldspathic Ceramics	Silica-based glass ceramics	Moderate strength, high translucency	Excellent aesthetics and optical properties	Veneers, anterior restorations	[20]
Lithium Disilicate Ceramics	Lithium disilicate glass-ceramic (Li_2_Si_2_O_5_)	Flexural strength ~360–400 MPa	High aesthetics, good durability	Crowns, veneers, inlays/onlays	[20,23]
Zirconia Ceramics (Y-TZP)	Yttria-stabilized zirconium dioxide	Very high strength (>900 MPa)	Exceptional fracture resistance	Posterior crowns, bridges, implant prostheses	[19,21]
Hybrid Ceramics (PICN)	Polymer-infiltrated ceramic network	Improved elasticity and fracture resistance	Reduced brittleness, better shock absorption	CAD–CAM restorations	[22]

**Table 4:** Mechanical properties of ceramic and hybrid restorative materials.

Material	Flexural Strength (MPa)	Elastic Modulus (GPa)	Fracture Toughness (MPa·m½)	References
Feldspathic ceramic	60–120	60–70	0.7–1.0	[20]
Lithium disilicate	360–400	90–100	2.5–3.0	[20,21]
Zirconia (Y-TZP)	900–1200	200–210	5–10	[19,21]
Hybrid ceramics (PICN)	150–200	25–35	1.5–2.0	[22]
Resin nanoceramic	150–250	12–15	1.2–1.8	[23]

**Table 5:** Emerging technologies and biomaterial approaches in contemporary restorative dentistry.

Technology/Material Type	Main Characteristics	Advantages	Clinical Longevity Evidence	Clinical Relevance	References
Nanostructured Resin Composites	Resin matrix with nanoscale fillers (silica, zirconia nanoparticles)	Improved polishability, wear resistance, aesthetic integration	Long-term clinical studies report survival rates comparable to conventional composites, with improved surface stability	Suitable for aesthetic restorations requiring enhanced surface quality and wear resistance, particularly in anterior and moderate-load posterior regions	[24,31]
Smart Bioactive Restorative Materials	pH-responsive materials releasing Ca^2+^, PO_4_^3−^ or F^−^ ions	Promote remineralization, reduce secondary caries risk, dynamic response to oral environment	Limited long-term clinical data; promising in vitro and short-term clinical studies	Recommended for patients with high caries risk or in minimally invasive approaches, where preventive and therapeutic effects are desired	[26,32]
Antimicrobial Nanocomposite Materials	Incorporation of antibacterial nanoparticles (Ag, ZnO) or antimicrobial monomers	Inhibition of bacterial adhesion and biofilm formation, prevention of recurrent caries	Mostly experimental or short-term clinical studies	Promising for reducing bacterial colonization and secondary caries, although clinical validation remains limited	[27,33]
CAD–CAM Restorative Materials	Digitally fabricated ceramics, hybrid ceramics and resin nanoceramics	High precision, reproducibility, improved marginal adaptation	Long-term clinical data available for zirconia and lithium disilicate restorations	Ideal for indirect restorations requiring high precision and durability, especially in digitally guided workflows	[23,34]
Artificial Intelligence in Biomaterial Design	Machine learning algorithms for predicting material performance and optimizing composition	Accelerated biomaterial development, predictive modeling of mechanical and biological properties	Emerging research field; limited direct clinical evidence	Useful for future personalized dentistry and predictive material selection, but currently limited to research and early clinical applications	[35,36]

## Data Availability

The information presented in this review was obtained exclusively from previously published scientific literature available in publicly accessible databases, including PubMed, Scopus, Web of Science, and Google Scholar.
